# Primary Small-Cell Neuroendocrine Carcinoma of the Vagina: A Case Report and Literature Review

**DOI:** 10.7759/cureus.95902

**Published:** 2025-11-01

**Authors:** Vishal Bahall, Lance De Barry, Triston De Barry

**Affiliations:** 1 Obstetrics and Gynaecology, The University of the West Indies, St. Augustine, TTO; 2 Obstetrics and Gynaecology, San Fernando General Hospital, San Fernando, TTO; 3 Internal Medicine, San Fernando General Hospital, San Fernando, TTO

**Keywords:** extrapulmonary neuroendocrine neoplasm, immunohistochemical diagnostic markers, ki-67 in neuroendocrine tumors, neuroendocrine tumor, primary vaginal cancer, small-cell neuroendocrine vaginal carcinoma

## Abstract

Small-cell neuroendocrine carcinoma (SCNC) of the vagina is an exceedingly rare and highly aggressive tumor of the female genital tract. The vast majority of SCNCs occur outside the female genital tract, with gynecological involvement being remarkably rare. Despite modern advances in tumor genomic profiling, SCNC continues to demonstrate an aggressive clinical course and remains associated with an unfavorable prognosis.

A 63-year-old woman presented to the Gynecology clinic with postmenopausal bleeding following a total abdominal hysterectomy with right salpingo-oophorectomy for uterine fibroids and a benign right ovarian cyst several years before. On assessment, a vaginal wall mass was discovered, and subsequent biopsy with histopathological assessment demonstrated an SCNC. Positron-emission tomography computed tomography confirmed primary vaginal SCNC with no metastatic foci. The patient subsequently underwent localized excision and was recommended for systemic treatment after a multidisciplinary team discussion.

Following the first reported case many years ago, only a small number of cases have been documented in the medical literature. Due to the paucity of publications on SCNC of the vagina, there is currently no consensus on standard treatment protocols for this tumor. Therefore, we aim to raise awareness and research on this unique presentation of SCNC of the vagina.

## Introduction

Neuroendocrine tumors of the female genital tract are a rare occurrence and present a critical clinical dilemma due to tumor heterogeneity and the lack of standardized treatment protocols. Small-cell neuroendocrine tumors of the vagina share clinical similarities with small-cell carcinoma of the lung, gastrointestinal tract, liver, prostate, and bladder [1]. Multiparous women appear to be more frequently affected, and the average age at onset is approximately 57 years [2].

The clinical manifestations of small-cell neuroendocrine carcinoma (SCNC) of the vagina are nonspecific, and most patients may report abnormal vaginal bleeding, contact bleeding, and abdominal or pelvic pain [3]. Small-cell neuroendocrine tumors can synthesize and secrete biologically active amines, neurotransmitters, paracrine factors, and hormones; rarely, because of this, patients may exhibit features of paraneoplastic syndrome [4].

Immunohistochemical assessment is required for the diagnosis of SCNC of the vagina [5]. Currently, there are no standardized treatment protocols, and management follows general guidelines for small-cell carcinomas at other sites. Patients are managed with a combination of surgical intervention followed by adjuvant chemoradiotherapy [6]. Emerging evidence indicates that SCNC of the vagina exhibits a notably aggressive clinical course, characterized by high metastatic potential and overall poor prognosis [7].

Herein, we describe the first case of SCNC of the vagina in the Caribbean. Our patient, a 63-year-old woman, presented with postmenopausal vaginal bleeding approximately 20 years after a total abdominal hysterectomy for uterine leiomyoma. On assessment, a vaginal mass was observed and biopsied. The histopathological evaluation demonstrated features consistent with SCNC, and follow-up imaging with positron emission tomography-computed tomography (PET-CT) confirmed this to be a primary lesion. The patient underwent localized reexcision of the vaginal mass, and subsequent systemic treatment was recommended after a multidisciplinary discussion of her treatment options.

## Case presentation

A 63-year-old woman presented to the Gynecology clinic with a history of postmenopausal bleeding for two weeks. She denied experiencing abdominopelvic pain, urinary or gastrointestinal symptoms, weight loss, night sweats, bloating, or indigestion. She underwent a total abdominal hysterectomy with right salpingo-oophorectomy approximately 20 years prior for a symptomatic uterine leiomyoma and a benign right ovarian cyst. The patient had a 15-year history of type II diabetes mellitus and chronic hypertension, managed with metformin and lisinopril, respectively. She reported no prior episodes of postmenopausal bleeding. She also had no personal or family history of malignancy.

On clinical examination, the abdomen was unremarkable. Vaginal and speculum examination demonstrated a typical, healthy-appearing vaginal vault with no evidence of any masses or contact bleeding. However, on the right posterior aspect of the vaginal wall, approximately 3 cm from the introitus, a 2 cm × 2 cm firm, nontender polypoid mass was observed.

**Figure 1:**
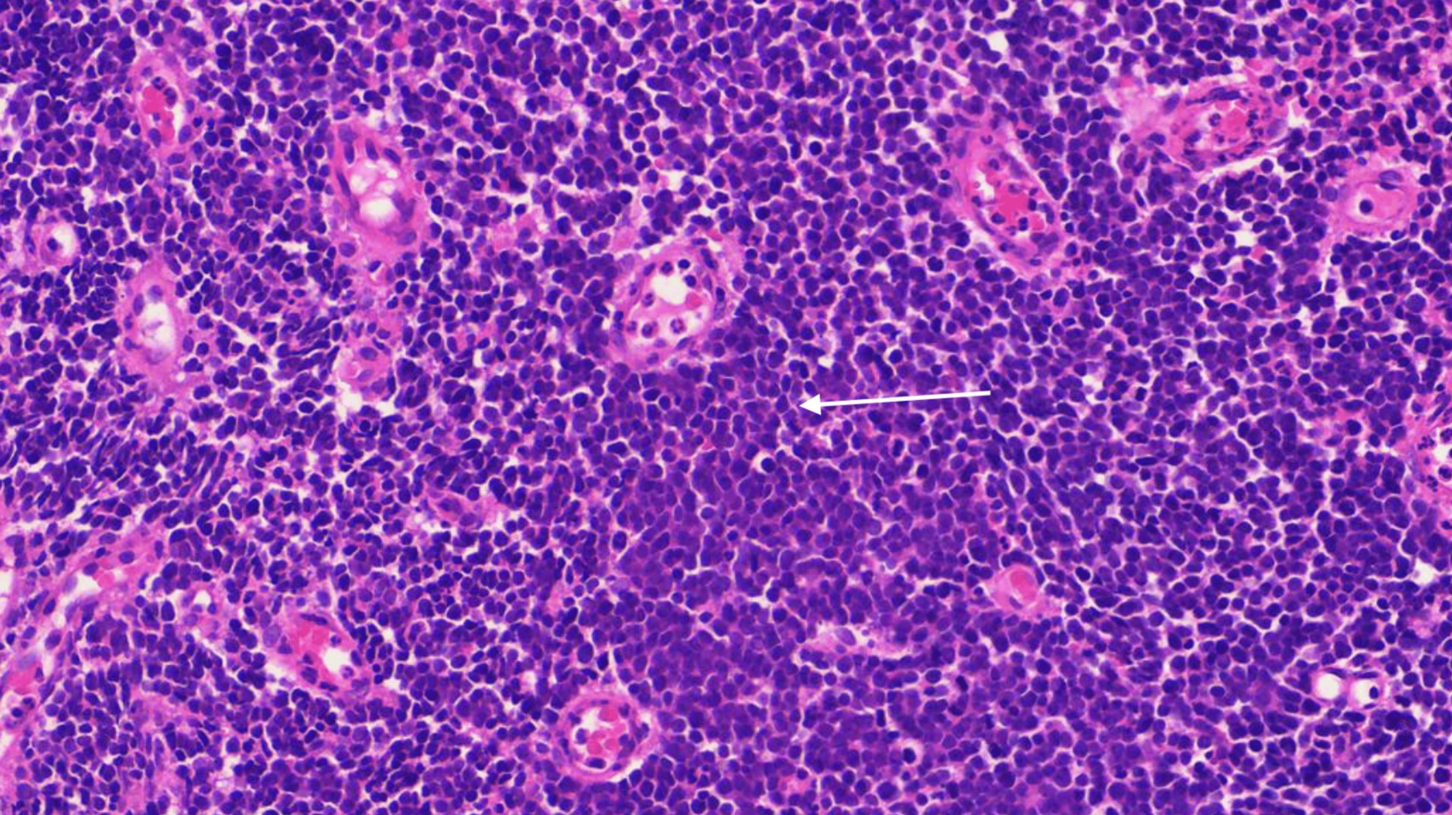
Histopathology demonstrating squamous mucosa with a nodule infiltrate of predominantly small to intermediate-sized lymphoid cells (white arrow) exhibiting fine granular chromatin

A biopsy of the vaginal wall lesion yielded a total tissue sample measuring 2.2 cm × 0.8 cm × 1.2 cm. Within this, a well-demarcated lesion measuring 0.7 cm × 0.6 cm × 0.3 cm was identified with a 0.1-cm clearance margin. Histologically, the lesion comprised squamous mucosa with a nodular infiltrate of predominantly small- to intermediate-sized lymphoid cells exhibiting fine granular chromatin (Figure 1).

Immunohistologically, the tumor cells were positive for the neuroendocrine markers synaptophysin and insulinoma-associated protein 1 (INSM1) and demonstrated an antigen Kiel 67 (Ki-67) proliferation rate of 100%. The tumor cells were negative for T- and B-lymphoid markers and chromogranin.

**Figure 2:**
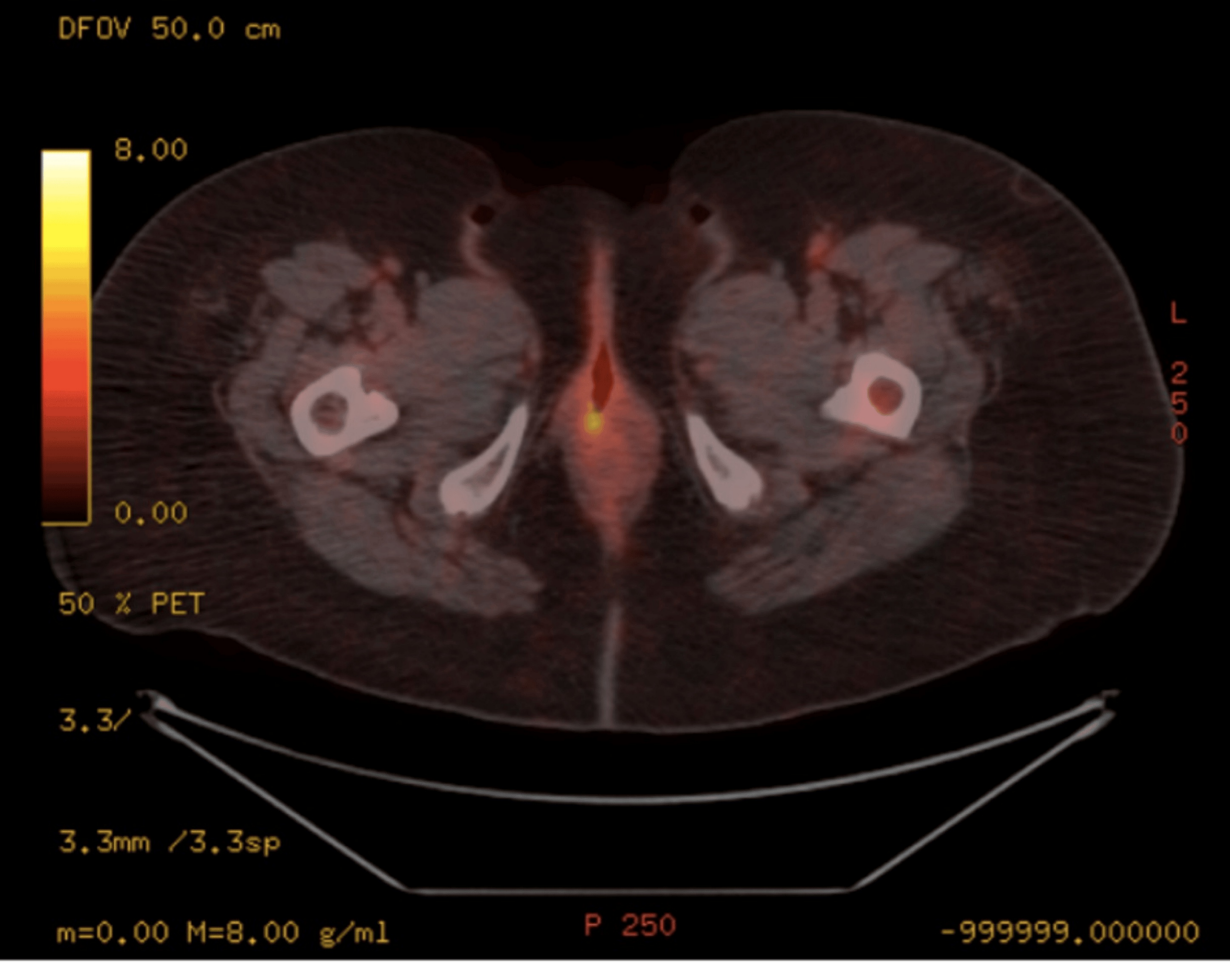
FDG and gallium-68 DOTATATE PET-CT demonstrating a small area of FDG accumulation in the right posterior wall of the vagina DFOV, dosimetric field of view; FDG, fluorodeoxyglucose; PET-CT, positron emission tomography-computed tomography

A fluorine-18 fluorodeoxyglucose (FDG) and gallium-68 DOTATATE whole-body PET-CT was arranged for neuroendocrine tumor staging (Figure 2). The scan demonstrated an area of FDG accumulation in the right posterior wall of the vagina, suggesting a primary focus on tumor cells and no evidence of metastatic disease. The case was discussed at the multidisciplinary team (MDT) meeting, and the patient was recommended to undergo a repeat localized excision of the vaginal mass with 1-cm margins. Histopathology demonstrated a benign epidermal inclusion cyst with no features of a neuroendocrine tumor.

Given the aggressive nature of SCNC, adjuvant chemotherapy was recommended following surgical management. Despite thorough counseling regarding the high risk of recurrence and the potential survival benefit of systemic therapy, the patient declined adjuvant treatment in favor of surveillance. This decision was made after careful consideration of the patient's preferences and overall clinical condition, reflecting a shared decision-making process. Unfortunately, the disease demonstrated rapid progression, and the patient succumbed within six months.

## Discussion

SCNC of the vagina is an exceptionally rare and aggressive malignancy [8]. Determining its exact incidence is challenging due to the scarcity of cases, with most available data coming from case reports and small case series [8]. In 1972, Albores-Saavedra reported the first case of SCNC in the female genital tract, specifically in the cervix, whereas in 1984, Scully et al. documented the first case of primary vaginal SCNC [9,10]. Epidemiological data indicate that only 5% of SCNCs occur in extrapulmonary sites, with just 2% involving the female genital tract [8]. SCNC predominantly affects the ovary and the cervix, while vulval and vaginal occurrences are even rarer [11]. Currently, SCNC is estimated to account for less than 1% of all vaginal malignancies [12]. The mean age at presentation is 57 years, and the average time from symptom onset to pathological diagnosis ranges from 1 to 54 months [2].

The pathogenesis of SCNC of the vagina remains unelucidated; however, high-risk human papillomavirus (HPV) subtypes may play a role in its development [13]. Kitazono et al. [14] were the first to report an association between high-risk HPV subtypes 16 and 18 and the development of small-cell carcinoma of the vagina in two cases. Similarly, a systematic review and meta-analysis of 403 cases by Castle et al. [15] found that 85% of cervical SCNC cases tested positive for HPV. The main viral oncoproteins of HPV (E6 and E7) promote rapid degradation of TP53 (tumor suppressor protein 53) and Rb1 (retinoblastoma transcriptional corepressor 1), driving malignant transformation and disrupting normal cell cycle regulation [13].

Epidemiological data suggest that HPV 18 exhibits a high affinity for glandular and neuroendocrine cells [16]. While HPV testing was not performed in our case, further research is needed to explore the etiological link between HPV 18 and SCNC of the vagina. In addition to HPV, other risk factors for primary vaginal SCNC include smoking, immunosuppression, older age, a history of cervical or vaginal dysplasia or cancer, and women of European descent [16].

Patients diagnosed with SCNC of the vagina commonly present with symptoms such as postmenopausal bleeding, a vaginal mass, dyspareunia, dysuria, or burning with micturition [12]. A minority of patients may exhibit no symptoms, but in rare cases, patients may display features of paraneoplastic syndrome [4]. Small-cell neuroendocrine tumors are composed of neuroendocrine cells capable of producing and releasing neurotransmitters, hormones, and paracrine factors with biological activity [4].

Cushing syndrome is the most common paraneoplastic syndrome associated with SCNC of the vagina, followed by hypercalcemia and syndrome of inappropriate antidiuretic hormone secretion [3]. In 1997, Colleran et al. were the first to report Cushing Syndrome caused by the ectopic production of adrenocorticotropic hormone from primary SCNC of the vagina [17]. Therefore, clinicians ought to display a high index of suspicion for vaginal SCNC when patients present with a vaginal mass, abnormal vaginal bleeding, and signs of paraneoplastic syndrome.

The diagnosis of SCNC of the vagina is confirmed on histopathology, and the findings are typically similar to those of primary pulmonary small-cell carcinoma [5]. Cells often display abundant nuclear chromatin, scant cytoplasm, an inconspicuous nucleolus that demonstrates diffuse solid growth, and neuroendocrine granules [18]. Moreover, immunohistochemistry may demonstrate positive stains for cytokeratin 20, cytokeratin (AE1/AE3 and CAM5.2), neuron-specific enolase, chromogranin A, and synaptophysin [5].

INSM1 is a novel immunostain for neuroendocrine tumors, specifically SCNCs of the genitourinary tract [19]. Antigen Kiel 67 (Ki-67), a prominent marker of cellular proliferation, is used for both diagnostic and prognostic purposes [20]. A high Ki-67 proliferation index indicates rapid cellular division, suggesting an aggressive tumor with a high likelihood of growth and metastasis [20]. In the case presented, immunohistochemistry demonstrated solid and diffuse staining for synaptophysin and INSM1, along with a remarkably high Ki-67 proliferation index approaching 100%, thus indicating an aggressive tumor with high metastatic potential.

Imaging modalities such as magnetic resonance imaging, computed tomography (CT), or PET-CT of the chest, abdomen, and pelvis are essential for delineating the extent of disease, assessing invasion into adjacent structures, detecting nodal or distant metastases, and planning treatment [12]. Fluorine-18 FDG PET is a widely used technique for staging and monitoring SCNC [12]. It has demonstrated greater sensitivity for detecting nodal involvement than CT alone, making it the preferred modality for assessing this type of cancer [12]. According to the National Comprehensive Cancer Network guidelines, lymph node involvement occurs in approximately 36% of cases, with distant metastases present in approximately 16% of cases [2].

Currently, no standardized treatment protocols exist for vaginal SCNC. Nevertheless, a multimodal approach combining surgery with adjuvant chemoradiotherapy appears to improve patient outcomes [6]. For early-stage disease confined to the upper third of the vagina, radical hysterectomy with pelvic lymph node dissection, along with partial or complete vaginectomy followed by adjuvant chemoradiotherapy, is recommended [7]. For disease localized to the lower vagina, local excision with 1-cm margins along with adjuvant chemoradiotherapy is the preferred approach [3]. Bilateral lymphadenectomy, including pelvic or groin lymph node dissection, may be warranted based on preoperative imaging findings [3].

Locally advanced disease, classified as T3-T4 and/or N1 M0 according to the tumor, node, and metastasis staging system, is typically managed with chemoradiation [3]. However, in cases with distant metastases, systemic chemotherapy is indicated. A standard regimen includes four to six cycles of combination chemotherapy [21]. Research suggests that combination chemotherapy improves survival outcomes compared with single-agent chemotherapy, even in early-stage disease [22]. The most widely used chemotherapy regimens include cisplatin with etoposide or a combination of cyclophosphamide, doxorubicin, and vincristine [21]. While precise dosage guidelines are scarce, Capote et al. recommend a regimen of cisplatin (60 mg/m^2^) on day 1 combined with etoposide (100 mg/m^2^) on days 1-3, administered every three weeks for up to six cycles, concurrent with pelvic radiation [3].

SCNC of the vagina is associated with a poor prognosis, and patients often encounter disease recurrence or progression [7]. Therefore, long-term follow-up is critical. The Society of Gynecologic Oncology guidelines for gynecologic SCNC recommend close surveillance, comprising vaginal, cervical, rectal, inguinal, and supraclavicular examinations, as well as PET-CT imaging. In cases of isolated local relapse, salvage surgery and/or radiotherapy followed by single-agent systemic chemotherapy with topotecan, paclitaxel, or docetaxel should be considered [23].

The decision to forego adjuvant chemotherapy in cases of SCNC of the vagina presents a significant clinical challenge, particularly due to the highly aggressive nature of this malignancy and its tendency for early metastasis. Adjuvant chemotherapy is typically recommended to enhance survival outcomes and reduce the risk of recurrence. In the absence of such treatment, close surveillance is critical, though it may offer limited protection against disease progression [24]. This approach highlights the importance of comprehensive patient counseling to ensure informed decision-making and vigilant monitoring for early signs of recurrence [24]. Unfortunately, due to the highly aggressive nature of SCNC, most patients presenting with relapse rapidly decline and eventually die as a result of distant metastases [7].

## Conclusions

SCNC of the vagina is an exceptionally rare and aggressive malignancy accounting for less than 1% of all vaginal cancers. Its pathogenesis is not yet fully understood, although high-risk HPV subtypes may be implicated in some cases. Patients often present with nonspecific symptoms and, rarely, paraneoplastic syndromes. An MDT is critical in managing patients presenting with primary vaginal SCNC. Histopathologists play an invaluable role in diagnosing this rare cancer, and novel immunohistochemical markers are now being used to improve diagnostic accuracy. Treatment involves a multimodal approach that combines surgery, chemotherapy, and radiotherapy; however, no standardized protocols exist. Despite aggressive therapies, patients often experience relapse, and most die within a year of diagnosis. Further research is required to improve the understanding and management of this rare malignancy.
